# Feasibility and Safety of Transfemoral Aortic Valve Replacement Using Local Anesthesia in the Catheterization Suite Versus General Anesthesia in the Operation Theater

**DOI:** 10.1002/ccd.70084

**Published:** 2025-08-08

**Authors:** Guillaume Abadié, Pierre Adrien Metzdorf, Giuseppe Lauria, Jeanne Varlot, Mazen Elfarra, Florian Eggenspieler, Juan‐Pablo Maureira, Batric Popovic, Edoardo Camenzind

**Affiliations:** ^1^ Department of Cardiology, University Hospital of Nancy University of Lorraine Nancy France; ^2^ Department of Cardiovascular Surgery and Transplantation, University Hospital of Nancy University of Lorraine Nancy France

**Keywords:** aortic stenosis, local anesthesia, percutaneous approach, transcatheter aortic valve implantation, transcatheter aortic valve replacement, transfemoral

## Abstract

**Background:**

For over a decade, transfemoral transcatheter aortic valve replacements (TF‐TAVR) have been performed at the University Hospital of Nancy. A minimalistic approach to TF‐TAVR, conducted without general anesthesia and entirely percutaneously in a catheterization suite (simplified approach), has been reported to be as safe and successful as the standard approach under general anesthesia in an operating room with surgical arterial cutdown. This study compares our initial experience of the simplified approach with the standard approach for TF‐TAVR.

**Aims:**

Assessing feasibility and safety of a simplified TAVR compared with the standard approach.

**Methods:**

We included TF‐TAVR performed from April 2020 to December 2021, and compared the simplified versus standard approach (SIMP‐group *n* = 89 vs. STD‐group *n* = 347). The endpoints included co‐primary endpoints defined by VARC‐3 (technical success at procedure exit, device success at discharge, and early safety at discharge), individual events during hospitalization, and mortality at 30 days and 1 year.

**Results:**

Baseline characteristics showed more comorbidities in the SIMP‐group. There were no significant differences for mortality rates and for the three co‐primary endpoints between the two groups. Bleeding and vascular complications were higher in the STD‐group (7.9% vs. 17%, *p* = 0.036 and 6.7% vs. 16%, *p* = 0.031). ICU and overall hospital stays were shorter in the SIMP‐group (median 1 vs. 2 days, *p* < 0.01 and 4 vs. 6 days, *p* < 0.001).

**Conclusion:**

This study suggests that the simplified approach for TF‐TAVR is as safe and successful as the standard approach, even for patients with greater comorbidities and operative risk. These findings could extend the simplified approach to younger, lower‐risk patients, optimizing hospital resource use and reducing waiting times for TAVR.

**Abbreviations**
ACT: activated clotting time
AVB: atrio‐ventricular block
Cath‐lab: catheterization laboratory
ESC: European society of cardiology
FPS: frame per second
ICU: intensive care unit
LAHB: left anterior hemi‐block
LBBB: left bundle branch block
PVR: para valvular regurgitation
RBBB: right bundle branch block
SAVR: surgical aortic valve replacement
TAVR: transcatheter aortic valve replacement
TF: trans‐femoral
TIA: transient ischemic accident
UH: university hospital
VARC‐3: Valve Academic Research Consortium (3rd edition in 2021)
VCA: vascular cerebral accident

## 1 Introduction

Since the first in‐man case in 2002, transcatheter aortic valve replacement (TAVR) has constantly evolved with a continuously increasing number of procedures per year reaching about 15,000 in France in 2020 [1]. During this time period, TAVR has progressed from a complex trans‐vascular to a progressively simplified percutaneous transfemoral (TF) approach facilitated also by the enduring improvement of transcatheter valve technologies [2]. In parallel to the procedural simplification of TAVR, progressively lower procedural complication rates were observed, and acute as well as long‐term outcomes have improved [3, 4]. This has allowed a gradual expansion of the indication of TAVR from surgically very high‐risk patients to lower‐risk patients [5, 6].

Several studies have described the safety and feasibility of exclusively percutaneous TF TAVR performed under local anesthesia in the catheterization suite, without the need of a dedicated anesthesiologist or of the use of transesophageal echocardiography [7]. This approach has demonstrated advantages on some clinical issues as the reduction of the need of catecholamines during the procedures, of delirium rates as well as of ICU and overall hospital stay [8]. Indeed, the progressive transition to lower‐risk patients as well as the technical improvements of the TAVR systems, has further facilitated this evolution.

In our center, TAVR has been performed initially with a “standard approach,” in an operating theater under general anesthesia. Since April 2020, a “simplified approach” has been adopted, and transfemoral transcatheter aortic valve replacements (TF‐TAVR) has been performed in the catheterization laboratory as described in previous studies [7, 9]. Patients were discussed at the weekly heart team staff meeting and were attributed either to a “simplified approach” in the catheterization suite or to a “standard approach” in the operating theater. The purpose of this study was the evaluation of both approaches relating to acute procedural safety and feasibility as well as to intra‐hospital outcomes and mortality up to 1 year follow‐up.

## 2 Methods

### 2.1 Patient Selection

This monocentric retrospective study included all consecutive TF‐TAVR performed at the University Hospital of Nancy from April 2020 to December 2021. Patients were discussed at the weekly heart team staff and were attributed either to a “simplified approach” in the catheterization suite or to a “standard approach” in the operating theater. Eighty‐nine patients were included in the “simplified approach” group (SIMP‐group) performed in the catheterization suite, and 347 patients in the “standard approach” group (STD‐group) performed in the operating theater. All patients had severe aortic stenosis (AS) according to the definitions of the 2017 and 2021 ESC guidelines [10, 11] including: (1) high gradient AS defined as mean gradient > 40 mmHg, peak velocity > 4 m/s, valve area ≤ 1 cm^2^ or ≤ 0.6 cm^2^/m^2^; (2) low flow low gradient AS defined as mean gradient < 40 mmHg, valve area ≤ 1 cm^2^, LVEF < 50%, and SVi ≤ 35 mL/m^2^; and (3) paradoxical low flow low gradient defined as mean gradient < 40 mmHg, valve area ≤ 1 cm^2^, LVEF ≥ 50%, SVi≤ 35 mL/m^2^. TAVR indications followed the ESC 2017 and 2021 guidelines.

Patients performed in the catheterization suite were excluded if: (1) TAVR access was not TF, (2) TAVR was combined with another procedure, and (3) the used valvular system was not the balloon expandable system from Edwards (Sapien, Edwards Lifesciences, Irvine, CA, USA) or the self‐expanding system from Medtronic (Evolut, Medtronic, Minneapolis, MN, USA), the only two systems used in the SIMP group.

TAVR under local anesthesia in the catheterization suite was performed only on high to very high‐risk, inoperable patients, who would not have been converted to open surgery if this had proven necessary. Those patients were referred to the “simplified approach” by the heart team after assessment of the preoperative risk using Euroscore 2 [12] as well as other clinical characteristics not taken into account in the Euroscore 2, such as femoral access evaluated by CT‐scan, compatibility with local anesthesia and conscious sedation, and acceptance by the patient. The type of valve was selected according to CT‐scan evaluation of the aortic valve, risk of annulus rupture and coronary status as well as ECG characteristics and the related risk of a pacemaker (PM) implantation.

### 2.2 Procedure

The “simplified approach” was performed in the catheterization suite under local anesthesia consisting in a 40 cc injection administered subcutaneously using a 1:1 mixture of 2% lidocaine (Aguettant, Lyon, France) and 7.5% ropivacaine (Aspen, Durban, South Africa) in the groin area. In some patients, on top of local anesthesia, hypnosis, or conscious sedation using Nalbuphine (Aguettant, Lyon, France, dose range from 5 to 10 mg IV) was used. The team included two interventional cardiologists, one X‐ray operator, and two nurses (total: *n* = 5). One of the nurses was in charge of the valve preparation. Antithrombotic treatments (anticoagulants and/or P2Y12‐inhibitors) were stopped 5 days before TAVR, and only Aspirin was maintained. After vascular access and before valve deployment, unfractionated heparin (UFH: 100 UI/kg) with a target ACT of 200−300 s and aspirin (Sanofi, Paris, France) (250 mg) were administrated IV. Initially, aortography was performed via a Pigtail catheter inserted via the contralateral femoral artery, then via the radial artery to minimize vascular‐ and bleeding‐complications. Femoral access was performed exclusively using a percutaneous technique, and the femoral artery was pre‐closed using initially two Proglide devices (Abbott, Chicago, IL, USA), followed by a single device which appeared as efficacious as the two Proglide technique [13, 14]. Rapid ventricular pacing was achieved via a temporary PM probe when the risk of complete AV‐block was considered high or via the intraventricular‐positioned Safari guidewire (Boston Scientific, Natick, USA). Sequential aortographies were performed to evaluate valvular positioning and aortic insufficiency post‐valve deployment. After removing the delivery system and the femoral valvular delivery sheath, a systematic abdominal aortography was performed following puncture site closure to exclude a vascular access complication. At the end of the procedure, protamine was administered in a 1:1 ratio to counteract the anticoagulant effect of UFH, and the nursing staff asked the patient to quantified maximally felt pain using the numeric rating scale (NRS) from 1 to 10 [15]. Temporary PM probe, when used for rapid pacing, was removed unless a persistent high‐degree conduction disorder was noted at the end of the procedure. Patients were monitored in the ICU for at least 24 h after the procedure.

The “Standard approach” took place in the operating theater, with a team including a cardiac surgeon, an interventional cardiologist, an anesthesiologist and nurse anesthetist, an X‐ray operator, and an operating theater nursing staff including 1 or 2 operating theater nurses (totally *n* = 6−7). TAVR was generally performed under general anesthesia, but some procedures, according to the anesthesiologist's judgment, were performed under conscious sedation without orotracheal intubation or laryngeal mask use. Very rarely was solely local anesthesia used. The vascular approach was either surgical or percutaneous, and pre‐closure was achieved using two Proglides in the percutaneous setting. The TAVR technique itself did not differ from the one of the SIMP‐group except that temporary probe, when used, was mostly kept in place at exit from procedure room.

### 2.3 Data Collection and Reported Events

The data collection took place from the TAVR procedure to hospital discharge using the data collected in the patient's computerized medical record (Dxcare, Dedalus, France). Mortality data were obtained via the medical records as well as using an online application by INSEE (French National Institute for Statistics and Economic Studies) (https://arbre.app/insee) gathering nationalwide death certificates [16].

All TAVR‐related cumulative events were informed and listed according to the predefined in‐hospital intervals (from entering procedure room up to university hospital discharge) as proposed by the VARC‐3 definitions [17]. The three hierarchical co‐primary endpoints according to VARC‐3 [17] were applied defining TAVR‐related success criteria (see Appendix for detailed definition).

Further procedural characteristics and length of in‐hospital stay as well as both 30 days and 1‐year all‐cause mortality have been reported.

### 2.4 Statistical Analysis

Qualitative variables were reported as frequencies and percentages; quantitative variables were reported as the mean ± standard deviation (SD) for normal distribution or as the median and interquartile range (IQR) for abnormal distribution. Comparisons between two qualitative variables were analyzed using the Fisher test or chi‐square. Comparisons between qualitative or quantitative variables were analyzed by Student's *t*‐test for normally distributed variables or Welch test as appropriate and by Mann–Whitney test for non‐normal distribution. Survival statistics were obtained using the Mantel−Haenszel log‐rank test. An *α* value of 0.05 was used for all analyses. Statistical analysis was performed using SPSS 20 software (SPSS Inc., Chicago, IL).

## 3 Results

**Figure 1:**
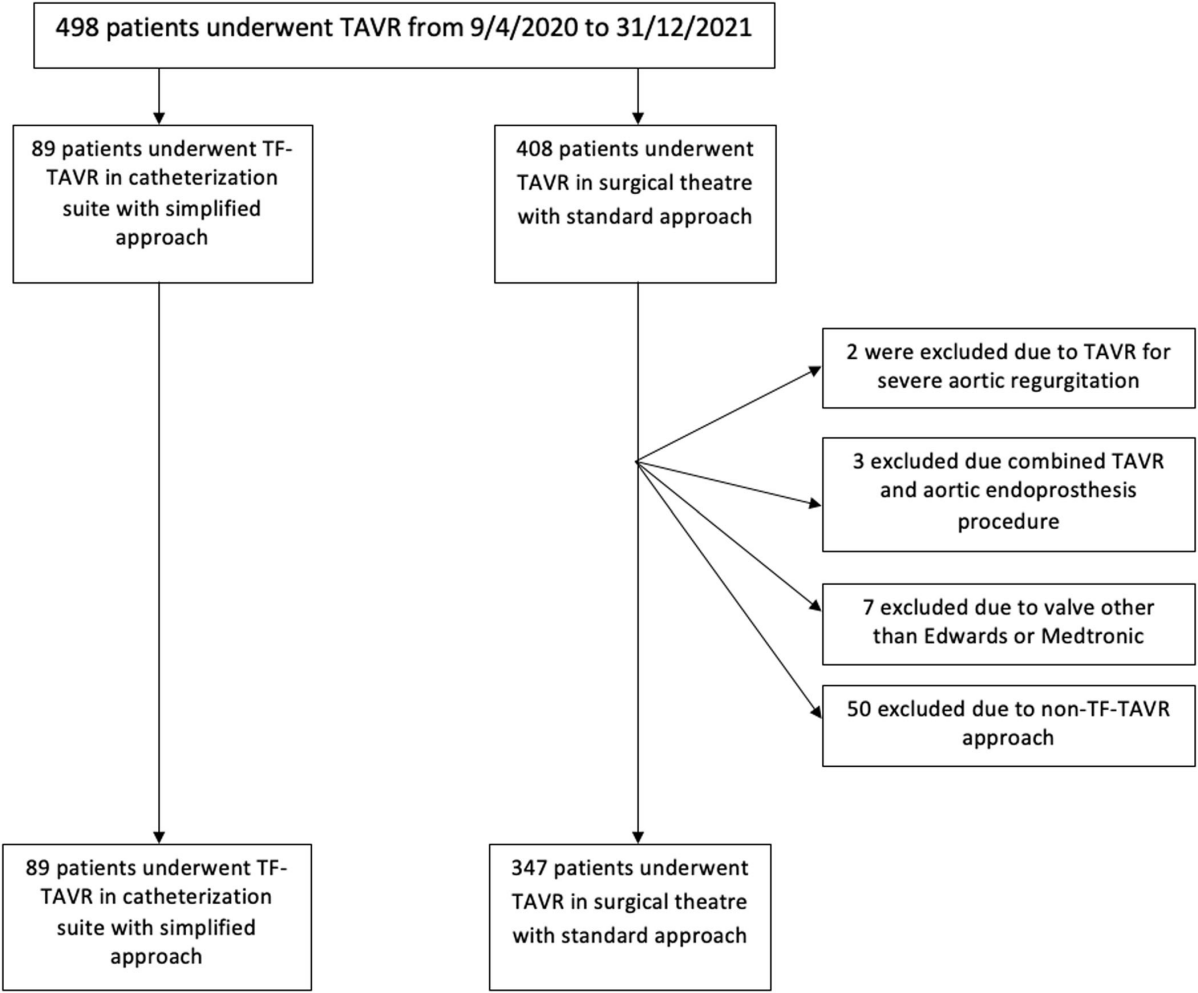
Flowchart of the included patients.

The flowchart of the enrolled patients is shown in Figure 1. Baseline characteristics of the patients allocated either to the “simplified approach” (*n* = 89; SIMP‐group) or to the “standard approach” (*n *= 347; STD‐group) are reported in Table 1. Patients in the SIMP‐group were significantly older (84.5 ± 5.2 vs. 81.5 ± 6.8, *p* < 0.001), had a higher operative mortality risk according to Euroscore 2 (3.6% vs. 3.0%, *p* = 0.01), a worse renal function (defined as CKD eGFR< 60 mL/min/1.73 m^2^: 67% vs. 53%, *p* = 0.017), and had more frequently hypertension. Further, they were more symptomatic (higher incidence of congestive heart failure and Heyden syndrome), had more often other significant valve disease, and had a lower LVEF. In the SIMP‐group, 25 patients (28.1%) had a Euroscore 2 between 4% and 8% (intermediate risk), and 17 (19.1%) had a score > 8% (high risk) versus in the STD‐group 90 patients (26%) with an intermediate risk and 33 patients (10%) with a high risk. Patients in the STD‐group had a higher BMI (28.1 ± 5.97 vs. 26.4 ± 3.73), were more often under P2Y12 inhibitors (31% vs. 13%), and had more frequently a previous PTCA (22% vs. 13%). Further, the mean aortic gradient was higher (48.4 ± 13.3 vs. 44.7 ± 12.8) and had a higher mean LVEF (58% vs. 55%).

**Table 1:** Baseline clinical and echocardiographic characteristics.

	Simplified group (*n* = 89)	Standard group (*n* = 347)	*p*
Age (years)	84.5 ± 5.2	81.5 ± 6.9	< 0.001
Female sex	41 (46%)	171 (49%)	0.59
Euroscore 2 mortality risk (%)	3.6 [2.50; 6.6]	3.0 [2.2; 5.0]	0.01
BMI (kg/m^2^)	26.4 ± 3.8	28.1 ± 6.0	< 0.001
Diabetes mellitus	23 (26%)	97 (28%)	0.69
Hypertension	82 (92%)	288 (83%)	0.032
P2Y12‐Inhibitors	13 (15%)	108 (31%)	< 0.01
Anticoagulant	42 (47%)	138 (40%)	0.2
Baseline pacemaker	22 (25%)	62 (18%)	0.14
COPD	6 (6.7%)	45 (13%)	0.1
Extracardiac arteriopathy^a^	13 (15%)	38 (11%)	0.34
Renal failure^b^	60 (67%)	185 (53%)	0.017
Previous cardiac surgery	6 (6.7%)	27 (7.8%)	0.74
Previous PTCA	15 (17%)	77 (22%)	< 0.01
Previous coronary bypass	5 (5.6%)	10 (2.9%)	0.31
Previous stroke/TIA	7 (7.9%)	38 (11%)	0.39
LVEF (%)	55.0 [40.0; 60.0]	58.0 [55.0; 61.5]	< 0.01
Mean aortic gradient (mmHg)	44.7 (12.8)	48.4 (13.3)	0.019
Other significative valve disease^c^	7 (7.9%)	8 (2.3%)	0.018
Symptoms			
Angina	10 (11%)	27 (7.8%)	0.3
Congestive heart failure	41 (46%)	54 (16%)	< 0.001
Heyde syndrome	4 (4.5%)	1 (0.29%)	< 0.01
NSTEMI	2 (2.2%)	2 (0.6%)	0.19
NYHA class ≥ II	85 (96%)	338 (97%)	0.31
II	14 (16%)	125 (36%)	< 0.001
III	57 (64%)	190 (55%)	0.11
IV	14 (16%)	23 (6.6%)	< 0.01
Syncope	9 (10%)	25 (7.2%)	0.36

*Note:* Data are expressed as mean ± standard deviation, number (%), or median [interquartile range].

Abbreviations: BMI, body mass index; COPD, chronic obstructive pulmonary disease; LVEF, left ventricle ejection fraction; PTCA, percutaneous transluminal coronary angioplasty; TIA, transient ischemic accident.

^a^ Euroscore 2 defined as carotid stenosis > 50%, planned or performed abdominal aortic surgery, limb arteries, or carotid arteries.

^b^ Defined as glomerular filtration rate < 60 mL/min/1.73 m^2^ of body surface area using Cockcroft−Gault formula.

^c^ Selected as other than aortic stenosis severe valve disease: Severe mitral regurgitation/stenosis and severe aortic regurgitation.

Overall cumulative events during the index hospitalization are listed in Table 2 and the segmentation according to site and time of occurrence in the Tables A1 and A2.

**Table 2:** Cumulative events from entry of procedure room to university hospital discharge.

	Simplified group (*n* = 89)	Standard group (*n* = 347)	*p*
Mortality	1 (1.1%)	3 (0.9%)	1
Neurologic events	3 (3.4%)	7 (2%)	0.43
Stroke	3 (3.4%)	3 (0.9%)	0.1
Mild NIHSS (from 5 to 15)	0 (0%)	2 (0.6%)	1
Minor NIHSS ( < 5)	3 (3.4%)	1 (0.3%)	0.028
TIA	0 (0%)	4 (1.2%)	0.59
Bleedings	7 (7.9%)	59 (17%)	0.032
Type 4	0 (0%)	0 (0%)	1
Type 3	1 (1.1%)	17 (4.9%)	0.14
Type 2	1 (1.1%)	15 (4.3%)	0.21
Type 1	5 (5.6%)	27 (7.8%)	0.49
Vascular complication	6 (6.7%)	54 (16%)	0.031
Major	3 (3.4%)	14 (4%)	1
Percutaneous intervention	2 (2.2%)	7 (2%)	1
Vascular surgery	1 (1.1%)	7 (2%)	1
Minor	3 (3.4%)	40 (12%)	0.021
Cardiac structural complication	1 (1.1%)	11 (3.2%)	0.47
Major	1 (1.1%)	10 (2.9%)	0.47
Conversion to open thoracic surgery	1 (1.1%)	6 (1.7%)	1
Percutaneous intervention	0 (0%)	2 (0.6%)	1
Surgical pericardial drainage	0 (0%)	2 (0.6%)	1
Minor	0 (0%)	1 (0.3%)	1
New conduction disturbances and arrhythmias	49 (55%)	174 (50%)	0.41
High degree conduction disturbance	19 (21%)	67 (19%)	0.67
Type 3 paroxysmal AVB	2 (2.2%)	17 (4.9%)	0.39
Type 3 permanent AVB	15 (17%)	46 (13%)	0.38
Other high‐degree conduction disturbance^a^	2 (2.2%)	4 (1.16%)	0.61
New LBBB	29 (33%)	95 (27%)	0.33
New onset AF or flutter	1 (1.1%)	12 (3.5%)	0.48
Permanent pacemaker implantation	22 (25%)	61 (18%)	0.13
Acute kidney injury (AKI)	2 (2.2%)	5 (1.5%)	0.64
AKI 3	0 (0%)	1 (0.3%)	
AKI 2	1 (1.1%)	1 (0.3%)	
AKI 1	1 (1.1%)	3 (0.9%)	
Paravalvular regurgitation (PVR)	9 (10%)	28 (8.1%)	0.54
Moderate to severe PVR ≥ 2	0 (0%)	0 (0%)	1
Mild PVR < 2	9 (10%)	28 (8.1%)	0.54
Valve malposition^b^ or 0/ > 1 valve used	1 (1.1%)	4 (1.2%)	1
0 valve in aortic position^c^	0 (0%)	2 (0.6%)	1
> 1 valve	1 (1.1%)	2 (0.6%)	1
Ectopic valve release	1 (1.1%)	3 (0.9%)	1

Abbreviations: AF, atrial fibrillation; AKI, acute kidney injury; AVB, atrioventricular block; LBBB, left branch bundle block; TIA, transient ischemic accident.

^a^ Includes: 3rd degree AVB (permanent or paroxysmic), syncopal 3rd degree sino‐atrial block, and LBBB/RBBB alternance.

^b^ Includes valve migration, embolization, or ectopic deployment. Three of those four patients had a 2nd valve used during the same procedure.

^c^ Includes one patient with one valve in ectopic position and one with zero valve deployed.

Table 2 shows a lower rate of bleedings and vascular complications in the SIMP‐group versus the STD‐group (7.9% vs. 17%, *p* = 0.036 and 6.7% vs. 16%, *p *= 0.031). The difference for the incidence of bleedings seems to be led by two types of bleedings (type 3: 1.1% vs. 4.9%, *p* = 0.14 and type 2: 1.1% vs. 4.3%, *p* = 0.21) and for vascular complications by minor vascular complications (3.4% vs. 12%, *p*= 0.021). New conduction disturbances were similar in both groups except for Type 3 permanent AVBs diagnosed from exit of procedure room to hospital discharge which were significantly more frequently observed in the SIMP‐group (9% vs. 1.4%, *p* < 0.01) (Table A2). Consequently, although not significantly, more PM were implanted in the SIMP‐group (25% vs. 18%, *p* = 0.13).

Other endpoints not included in VARC‐3 definitions are listed in Table 3. Concerning the procedure itself, the only detectable difference was related to the amount of X‐ray delivered which was slightly lower in the catheterization room. Concerning the overall in‐hospital length of stay (LOS) (including both university hospital and peripheral centers), patients in the SIMP‐group had a shorter stay than in the STD‐group with a median of 4 versus 6 days (*p* < 0.001). For the solely LOS at the university hospital, the median total length was similar (4 days), with however a shorter stay in the ICU for the SIMP‐group.

**Table 3:** Endpoints not included in the Valve Academic Research Consortium 3 endpoints.

	Simplified group (*n* = 89)	Standard group (*n* = 347)	*p*
Valve brand^a^			0.58
Sapien (Edwards)	64 (72%)	264 (76%)	
Evolut (Medtronic)	25 (28%)	80 (23%)	
TAVR procedure duration (min)	48.0 [42.0; 57.0]	51.0 [41.0; 62.0]	0.077
Fluoroscopy time (min)	10.1 [8.07; 14.4]	10.5 [8.10; 14.3]	0.75
X‐ray delivered (Gy/cm^2^)	36.23 [25,0; 58,6]	47.6 [26,3; 76,3]	0.04
Iodine amount (cc)	117 [90.0; 151]	115 [87.0; 150]	0.54
Pain^b^	3 [2; 5]	NA	
LOS in ICU after TAVR (days)	1 [1; 2]	2 [1; 4]	< 0.01
LOS at university hospital after TAVR (days)^c^	4 [3; 6]	4 [2; 6]	0.98
LOS overall in‐hospital after TAVR (days)^d^	4 [3; 6]	6 [4; 8]	< 0.001
Early discharge^e^ from UH hospital stay	38 (43%)	110 (32%)	0.05
Early discharge^e^ from overall hospital stay	35 (39%)	38 (11%)	< 0.001

*Note:* Data are expressed as median [interquartile range], and number (%).

Abbreviations: ICU, intensive care unit; LOS, length of stay; UH, university hospital.

^a^ *n* = 431, due to failure to perform TAVR.

^b^ Numeric rating scale, from 0 to 10, assessed during the procedure.

^c^ LOS at the university hospital in ICU and the conventional care unit.

^d^ Overall LOS at university hospital and peripheral center.

^e^ Early discharge: hospitalization < 4 days.

Table 4 shows the three co‐primary endpoints. Technical success rate at the exit of the procedure room did not show any significant difference between SIMP‐group and STD‐group (97% vs. 94%, *p* = 0.59). Device success at discharge was also similar between the two groups (80% vs. 78%, *p* = 0.78) as was early safety at discharge (61% vs. 67%, *p* = 0.27).

**Table 4:** Co‐primary endpoints according to Valve Academic Research Consortium 3.

	Simplified‐group (*n* = 89)	Standard group (*n* = 347)	*p*
Technical success at exit from procedure room^a^	86 (97%)	327 (94%)	0.59
Device success at discharge^b^,^d^	71 (80%)	272 (78%)	0.78
Early safety at discharge^c^,^d^	54 (61%)	232 (67%)	0.27

*Note:* Data are expressed as mean ± standard deviation, median [interquartile range], and number (%).

^a^ Valve Academic Research Consortium 3 (VARC3) defined “technical success at exit from procedure room”: composite of freedom from mortality, successful access/delivery of the device/retrieval of the delivery system, correct positioning of a single prosthetic heart valve into the proper anatomical location and freedom from surgery or intervention related to the device or to major vascular or access‐related or cardiac structural complication.

^b^ VARC3 defined “in‐hospital device success”: Composite of technical success, freedom from mortality at discharge, freedom from surgery or intervention related to the device or to major vascular or access‐related or cardiac structural complication, and intended performance of the valve (mean gradient < 20 mmHg, peak velocity < 3 m/s, and less than moderate PVR).

^c^ VARC3 defined “early safety at discharge”: Composite of freedom from all‐cause mortality/from all stroke/from VARC type 2–4 bleeding/from major vascular, access‐related, or cardiac structural complication/from acute kidney injury stage 3 or 4/from moderate or severe PVR/from new permanent pacemaker due to procedure‐related conduction abnormalities and freedom from surgery or intervention related to the device.

^d^ Discharge from university hospital.

**Figure 2:**
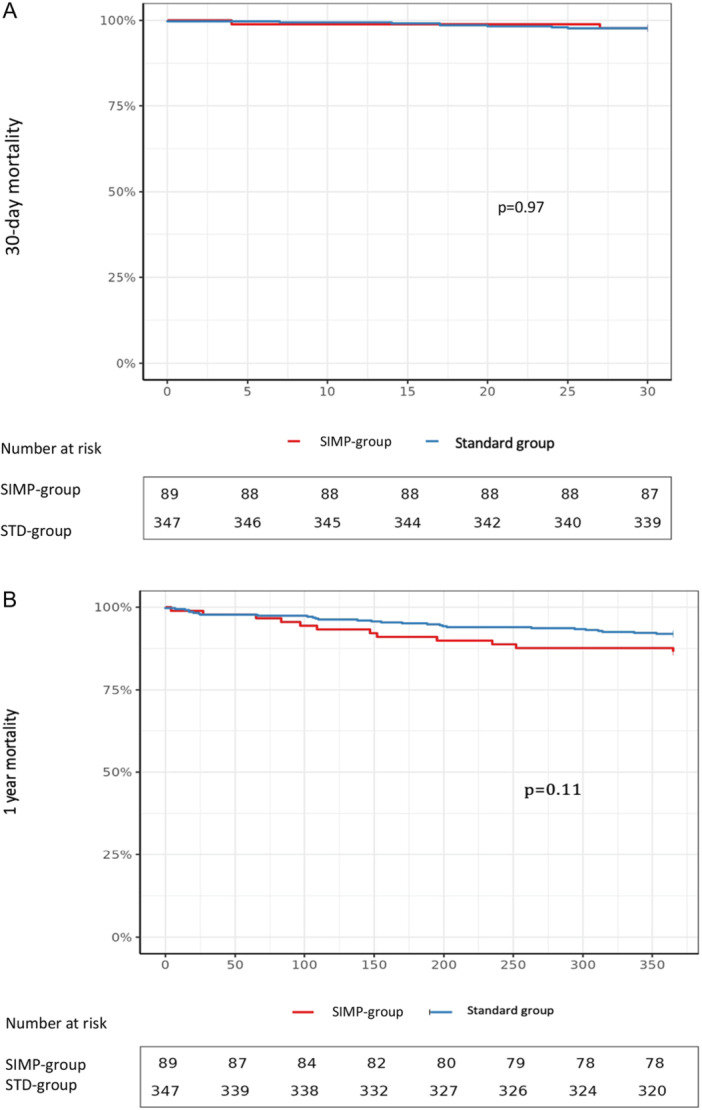
30 days and 1 year survival curves between the SIMP‐group and STD‐group using the log‐rank test. [Color figure can be viewed at wileyonlinelibrary.com]

The survival curves at 30 days and 1 year were not significantly different between the two groups (Figure 2a,b).

## 4 Discussion

The main findings of the current monocentric evaluation of trans‐femoral TAVR performed with local anesthesia in the catheterization laboratory (SIMP‐group) versus general anesthesia in the operating theater (STD‐group) showed that: (1) Intra‐hospital, 30 days and 1 year mortality were similar, (2) “early safety at discharge” according to VARC‐3 was not significantly different, (3) among the single cumulative events during the index hospitalization bleedings and vascular complications were significantly more frequent in the STD‐group, (4) among the single cumulative outcomes “from exit procedure room to hospital discharge” high degree conduction disturbances were significantly more frequent in the SIMP‐group engendering more PM implantations, and (5) ICU discharge and overall duration of hospitalization was shorter in the SIMP‐group.

The study period from April 2020 to December 2021 coincided with the Covid‐19 pandemic which had two peaks in the North‐Eastern region of France (October 2020 and March/April 2021). During this period, due to the backlog of patients needing TAVR, no influence on recruitment nor on the patient's baseline characteristics was observed in either arm.

Incidence of mortality was similar among the two groups during the index hospitalization for TF‐TAVR (1.1% vs. 0.9%, *p* = 1; Table 2) and slightly lower than in previous series (1.7% in Wienemann et al., 1.6% in Saia et al., and 3.4% in Husser et al.) [18, 19, 20]. Thirty‐day mortality was 2.2% in the SIMP‐group versus 2.3% in the STD‐group, and 1‐year mortality reached 13.5% versus 8.1%, respectively (Figure 2). The current outcomes compare favorably to a previous large cohort of Husser et al. reporting a mortality of 4.2% at 30 days and 16.7% at 1 year [20]. The tendency of a lower 1‐year survival in the SIMP‐group despite a numerically better 30 days survival rate may be explained by the fact that patient were significantly older and symptomatic, had a more advanced kidney dysfunction, and a lower LVEF (Table 1) [21, 22]. However, a potential influence of COVID on mortality cannot be ruled out due to an observed 1.5 excess in the mortality index touching in particular the population over 70 in this region, despite the fact that mortality figures post‐TAVR were low in both series compared to the literature.

The three co‐primary endpoints reflecting a hierarchical event‐free procedural success and follow‐up up to hospital discharge, as termed according to its components in the VARC‐3 definitions [17], did not reveal any significant difference between the two groups (Table 4). Technical success at exit from procedure room were consistent with the series of Wienemann et al. and of Doldi et al. with a reported technical success rate of 94.3% and 91.8% [19, 23]. Technical success according to the VARC‐3 definition has shown to be an important prognostic factor with a twofold increase of a composite endpoint of “1‐year mortality and stroke” after TAVR when technical success was not achieved (11.5% vs. 3.5%, *p* < 0.001) [24]. In the current study, technical success was similar in both groups and, thus, not surprisingly, also 1 year prognosis.

The cumulative events during the index hospitalization (Table 2) show a similar incidence of neurologic events, a composite of stroke and TIA, in both groups (3.4% vs. 2%, *p* = 0.25), and of a similar magnitude as reported in the literature (ranging from 2% to 5%) [25, 26, 27, 28, 29]. Of the four TIA in the STD‐group, three happened within the 24 h following the procedure, and out of them, one was diagnosed directly at recovery from anesthesia. The last one occurred on Day 3 after TAVR. However, incidence of strokes was numerically higher in the SIMP‐group (3.4% vs. 0.9%, *p* = 0.1). A large meta‐analysis and review suggested that chronic kidney disease was a major predictive risk factor for stroke after TAVR [30, 31], a morbidity that prevailed significantly in the SIMP‐group but this may not explain by itself the observed difference. Of interest is that TF‐TAVR under local anesthesia enables a more accurate neurological follow‐up of the patient during the procedure as well as an earlier diagnosis of neurological events already in the procedure room. Under general anesthesia, TIA are likely to be under‐estimated and strokes only diagnosed after the patient's wake‐up, as observed in the current study. A further explanation may be that patients in the SIMP group were under mono‐antiplatelet therapy solely, and in the STD group, more frequently under dual antiplatelet therapy on top of full‐dose UFH therapy during the procedure; UFH therapy, which was systematically reversed at the end of the procedure in both groups.

Concerning bleedings, no type 4 bleedings were observed in the current series, and type 3 bleedings were similar in both groups, although more frequent in the STD‐group (1 [1.1%] vs. 17 [4.9%], *p* = 0.14). In the SIMP‐group, one patient with annulus rupture and tamponade also experienced a drop of ≥ 5 g/dL in hemoglobin needing multiple transfusions. In the STD‐group, 17 patients developed a type 3 bleeding secondary to tamponades (annulus rupture [*n* = 2] or LV laceration [*n* = 7]) and eight secondary to puncture site complications (five artery lacerations treated surgically [*n* = 4] or percutaneously [*n* = 1], two major scarpa hematoma and one retroperitoneal bleeding; these three last complications have been treated conservatively). Among the vascular complications and cardiac structural complications converted to open thoracic surgery, six patients needed multiple red blood cell transfusions ( > 2 RBC) for a hemorrhagic shock. The current study compares favorably to a recent study by Avvedimento et al., describing a 5.4% incidence rate of type 3 bleedings in a larger cohort including 2384 patients, of which 761 (31.9%) had at least one type 1 to type 4 bleeding complication according to VARC3 criteria [32].

Type 2 bleedings incidence were also similar in both groups (1.1% [*n* = 1] in SIMP group and 4.3% [*n* = 15] in STD group, *p* = 0.21) and mostly engendering anemia requiring two to four RBC transfusions. Type 2 bleedings were less frequent in the current series than in the series of Avvedimento et al. reporting 15.1% of type 2 bleedings during hospitalization [32].

In the literature, type 2−4 bleedings are frequently pooled as in the VARC‐3 co‐primary endpoints. In this study, pooled data of type 2−4 bleedings showed a significantly lower bleeding incidence in the SIMP‐group (2.2% vs. 9.2%, *p* = 0.023). This difference may be explained by several factors. All accesses were percutaneous in the SIMP‐group and 19 patients had a surgical cut down of the common femoral artery in the STD‐group. The extent of femoral calcifications, artery size, and tortuosity were specifically collected data pre‐procedure, and tricky vascular accesses would sometimes lead to referring the patient to the “standard approach.” Further, the incidence of DAPT was higher in the STD‐group.

Major vascular complications did not differ among the two groups. However, minor vascular complications, which may seem irrelevant, were more frequent in the STD‐group and observed in 3.4% in the SIMP‐group and 12% in the STD‐group (*p* = 0.021). A recent study including 2161 patients showed that minor vascular events, with a reported incidence of 13% in their series, increased operative time, operative and in‐hospital mortality, hospital LOS, and impacted lastly 5‐year mortality [33]. In the current series, there was no difference in in‐hospital outcome or mortality according to the presence of minor vascular complications. Vascular complications can be partially explained by the height of the puncture site, the presence of arterial calcifications, and the type of access (percutaneous vs*.* surgical). Further, some studies showed that female sex and sheath‐to‐femoral‐artery‐ratio were independent predictors of vascular events [34, 35]. Systematic ultrasound‐guided puncture can reduce some of these complications by puncturing the front side of the common femoral artery, avoiding the bifurcation of superficial and deep femoral artery as well as calcifications, which can decrease the closing systems efficiency [36, 37]. Systematic ultrasound guidance for puncturing the artery was used in the current study in both groups, and percutaneous (pre)closure was performed using the Proglide Perclosure systems (Abbott, Chicago, IL, USA) in both groups.

Cardiac structural complications did not differ between the two groups (1.1% vs. 3.2%, *p* = 0.47). The SIMP‐group compares favorably to the literature reporting an incidence rate of 1.17% in a very large cohort of Pineda et al. and of 2.5% in a series of Liang et al. [38, 39]. In the STD‐group, a higher incidence was observed that may be attributed to a higher pool of physicians performing TAVI (*n* = 2 vs. *n* = 8)

ECG changes and in particular new conduction disturbances are a major concern after TAVR (Table 2). Prior TAVR type I AVB and left anterior hemiblock tended to be more frequent in the SIMP‐group (Table A3). The most frequently acquired conduction abnormality in the current two series was a new‐onset of LBBB, followed by permanent 3rd degree AVB and by paroxysmal 3rd degree AVB. Allover, new conduction disturbances were observed in 54.4% of the cases in the SIMP‐group (LBBB 33%, type 3 AVB 19.2%, other 2.2%) and in 46.1% of the cases in the STD‐group (LBBB 27%, type 3 AVB 17.9%, other 1.2%). Of all patients, 25% in the SIMP group and 18% in the STD group benefited from a permanent PM implantation. Permanent PM after TAVR were higher in the SIMP‐group and higher than in most randomized as well as real‐life studies [7, 8, 9, 25, 40, 41] or real‐life registry like FRANCE 2 or FRANCE TAVR [4]. Recommendations for PM implantation after TAVR have evolved and have become more strict between the 2018 AHA guidelines [42] and the 2021 ESC guidelines [43]. Prior to 2021, AHA recommendations suggested implantation of a PM for a new persistent LBBB, and since 2021, those patients should have had prior an electrophysiological exploration. Nine patients in this study (3.4% in SIMP‐group and 1.7% in STD‐group) had a PM implantation for a new persistent LBBB (QRS > 150 ms), when according to 2021 ESC recommendations, an electrophysiological exploration would have been indicated. In the current study, patients who had a PM implantation for paroxysmal 3rd degree AVB without prior RBBB had their TAVR procedure in 2020 or early 2021. In the SIMP‐group, PM implantation decreased from 36% of TAVR in 2020 to 20% in 2021 which is closer to the literature, even if it remains slightly higher [8]. To be mentioned that no difference was observed in the used type of valve‐systems in both groups (self‐expandable vs. balloon expandable). In the current series and in both groups, new conduction disturbances were more after balloon expandable valve deployment (Table A3).

Acute kidney injury (AKI) was low in both groups (2.2% vs. 1.44%, *p* = 0.59) and lower than in a previous series from Bagur et al. (AKI allover in 11.7% of the cases and 1.4% needed dialysis) [44].

Paravalvular regurgitation ≥ 2 is a prognostically important variable after TAVR as shown in the PARTNER 2 trial [45] with a 2.85 higher death rate at 2 years for severe or moderate aortic regurgitation versus mild regurgitation or trace/none. Paravalvular mild (grade < 2) regurgitation occurred similarly in both groups (10% vs. 8.1%, *p* = 0.51) and numerically close to the series of Gilard et al. (grade ≥ 2: 14.7%) [46] but lower than in the series of Jabbar et al. (35% had at least mild aortic regurgitation) [47].

Valve malposition frequency and impact on 1‐year mortality in the global population compared with optimal positioning (20% vs. 9.1%, *p* = 0.38) was similar with a previous serie from Kim et al. (respectively 0.92% and 18%) [48].

Different evaluation criteria are not detailed in the VARC 3 definition that are of interest in this comparison and listed in Table 3.

The radiation dose was higher in the STD group despite similar fluoroscopy times. This can be due to the different models or time of life of the used angiographic systems (Siemens Artis‐Q, Erlangen, Germany 3 years old in the catheterization suite vs. Siemens Artis Zeego, Erlangen Germany 12 years old in the operating theater), to the fluoroscopic and angiographic image acquisition settings (same baseline set‐ups: fluoroscopy and angiography: 7.5 fps at both sites) or to a larger use of cine‐angiography versus fluoroscopy during the procedure. Fluoroscopy time in the current study was shorter for both groups (overall 10.4 min) as compared to a previous study of Attizzani et al. (17 min in the local anesthesia group and 19 min in the conscious sedation group). In that very same study, the iodine amount was comparable with the current series (local anesthesia: 134 mL and conscious sedation: 83 mL vs. current series overall 115 mL) [49], but lower than in other series (Serletis‐Bizio et al.: 163 mL and Babalarios et al.: 126 mL) [41, 50].

Pain assessment in the SIMP‐group was performed using the NRS. The median NRS pain was 3.00 [2.00; 5.00] on a scale from 0 to 10, which corresponds to a low level of pain [15, 51]. However, it remains a subjective assessment, and indeed it cannot be compared with the STD‐group, but it revealed an acceptable intensity of pain during the procedure using a local anesthesia 40 cc of a 1:1 mixture of 2% lidocaine and 7.5% ropivacaine.

Although the median total length of university hospital stay was similar for both groups, ICU stay was significantly shorter in the SIMP‐group (1 vs. 2 days, *p* < 0.1; Table 3). In the university hospital, the SIMP‐group was monitored in the cardiac ICU, whereas the STD‐group in the cardiac surgery ICU. The difference in LOS in the ICU may be explained by the management of vascular and bleeding complications which were more frequent in the STD‐group. Also, the habits to manage acute high‐degree conduction disorder and temporary PM may differ between the two ICUs and lead to variable monitoring periods. Moreover, TAVR were performed 5 days a week in the SIMP‐group and 2 days a week in the STD‐group and elective permanent PM implantation was performed 3 days a week. Thus, the waiting time for a permanent PM may have been longer in the STD group. The LOS in the ICU for the SIMP‐group was equal to the series of Babalarios et al. (1 day) [41], and equal for both the SIMP‐group and STD‐group in the meta‐analysis of Villablanca et al. [52]. Overall median in‐hospital LOS, including university hospital and peripheral hospital, was significantly shorter in the SIMP‐group versus the STD‐group (4 days [3; 6] vs. 6 days [4; 8]; *p* < 0.001). This may be due to diverse rehabilitation programs in different peripheral centers, or may be secondary to vascular access technique (surgical cut down) or complications, which were higher in the STD‐group. The current overall median hospital durations were comparable to the literature (5 days for percutaneous‐only procedures and 6 days for regular approach in a series from Denimal et al.; 7 days in the meta‐analysis from Villablanca et al.) [7, 52].

Of interest is that recent studies have shown that an early discharge does not increase mortality or readmission rate at 30 days and even reduce long term readmission rate [53, 54]. In our study, overall median length of hospital stay (university hospital and eventual peripheral centers), was less than 4 days for 39% of the patients in the SIMP‐group and 11% of the patients in the STD‐group.

Our study has several limitations. As with any retrospective single‐center study, the current results must be evaluated in the perspective of the quality and reliability of the available data collected from the computerized records. In addition, certain confounding factors may have been overlooked, notably the absence of complete CT‐scan data in the STD group due to the fact that they have been partially performed in private clinics. Also, some procedural informations have not been systematically recorded as balloon pre‐dilatation before valve deployment or balloon post‐dilation post valve deployment, which may have influenced the incidence of certain events. Although our population is larger than in other published TAVR‐related series, it remains a relatively small collective which may present a risk of statistical bias.

As a consequence of this evaluation showing a very similar safety outcome of TAVR independently of the site and modality of TAVR realization, TAVR is now performed in the catheterization suite as well as in the operating theater, and cardiovascular surgery is on standby for the cases performed in the catheterization suite.

## 5 Conclusion

This study demonstrated that a simplified TF‐TAVR approach using local anesthesia in the catheterization suite was feasible and safe even in very high‐risk/inoperable patients and enabled an equivalent technical success, a short hospital stay without higher adverse events as compared to the standard approach with anesthesia/sedation in the operation theater. These results will make it possible to extend the simplified approach TF‐TAVR to younger, lower‐risk patients to increase TF‐TAVR capacities, making better use of in‐hospital resources and abolish waiting times for TAVR.

## Ethics Statement

This is an observational study. The local Research Committee of Nancy University Hospital has confirmed that no ethical approval is required. This paper is not under review or published elsewhere.

## Consent

Informed consent was obtained during each patient's hospital stay, and information was given about the use of their data for research.

## Conflicts of Interest

The authors declare no conflicts of interest.

## 1

**VARC‐3 Composite Endpoints**:

− Technical success at exit from procedure room: composite of freedom from mortality, successful access/delivery of the device and retrieval of the delivery system, correct positioning of a single prosthetic heart valve into the proper anatomical location, and freedom from surgery or intervention related to the device or to major vascular or access‐related or cardiac structural complication.
− In‐hospital device success: composite of technical success, freedom from mortality at exit, freedom from surgery or intervention related to the device or to major vascular or access‐related or cardiac structural complication, and intended performance of the valve (mean gradient < 20 mmHg, peak velocity < 3 m/s, and less than moderate PVR)
− Early safety at discharge including freedom from: all‐cause mortality, from all stroke, from VARC type 2–4 bleeding, from major vascular, access‐related, or cardiac structural complication, from acute kidney injury stage 3 or 4, from moderate or severe PVR, from new permanent pacemaker due to procedure‐related conduction abnormalities and from surgery or intervention related to the device.

**Table A1:** Cumulative events recorded from enter to exit procedure room.

	Simplified group (*n* = 89)	Standard group (*n* = 347)	*p*
Mortality	0 (0%)	1 (0.3%)	1
Neurologic events	1 (1.1%)	NA	NA
Stroke	1 (1.1%)	NA	NA
Minor NIHSS ( < 5)	1 (1.1%)	NA	NA
TIA	0 (0%)	NA	NA
Bleeding			
Type 4	0 (0%)	0 (0%)	1
Type 3	1 (1.1%)	12 (3.5%)	0.48
Type 2	0 (0%)	4 (1.2%)	0.59
Type 1	0 (0%)	8 (2.3%)	0.37
Vascular complication			
Major	1 (1.1%)	10 (2.9%)	0.47
Percutaneous intervention	1 (1.1%)	6 (1.8%)	1
Vascular surgery	0 (0%)	4 (1.2%)	0.59
Minor	0 (0%)	15 (4.3%)	0.049
Cardiac structural complication			
Major	1 (1.1%)	8 (2.3%)	0.69
Conversion to open thoracic surgery	1 (1.1%)	6 (1.7%)	1
Percutaneous intervention	0 (0%)	2 (0.6%)	1
Minor	0 (0%)	1 (0.3%)	1
Valve‐related complication			
Implantation of multiple transcatheter valves during the index hospitalization	1 (1.1%)	3 (0.9%)	1
Valve mispositioning	1 (1.1%)	3 (0.9%)	1
New conduction disturbances and arrhythmias			
High degree conduction disturbance			
Type 3 paroxysmal AVB	2 (2.2%)	14 (4%)	0.54
Type 3 permanent AVB	7 (7.9%)	41 (11.8%)	0.29
Other high‐degree conduction disturbance^a^	0 (0%)	2 (0.6%)	1
New left bundle branch block	29 (33%)	95 (27%)	0.33

Abbreviation: NA, not applicable.

^a^ includes: 3rd degree AVB (permanent or paroxysmic), syncopal 3rd degree sino‐atrial block, and LBBB/RBBB alternance.

Table A1

**Table A2:** Cumulative events recorded from exit of procedure room to university hospital discharge.

	Simplified group (*n* = 89)	Standard group (*n* = 347)	*p*
Mortality	1 (1.1%)	2 (0.6%)	0.5
Neurologic events	2 (2.2%)	7 (2%)	1
Stroke	2 (2.2%)	3 (0.9%)	0.27
Mild NIHSS (from 5 to 15)	0 (0%)	2 (0.6%)	1
Minor NIHSS ( < 5)	2 (2.2%)	1 (0.3%)	0.11
TIA	0 (0%)	4 (1.2%)	0.59
Bleeding			
Type 4	0 (0%)	0 (0%)	1
Type 3	0 (0%)	5 (1.4%)	0.59
Type 2	1 (1.1%)	11 (3.2%)	0.47
Type 1	5 (5.6%)	19 (5.5%)	1
Vascular complication			
Major	2 (2.2%)	4 (1.2%)	0.61
Percutaneous intervention	1 (1.1%)	1 (0.3%)	0.37
Vascular surgery	1 (1.1%)	3 (0.9%)	1
Minor	3 (3.4%)	25 (7.2%)	0.19
Cardiac structural complication			
Major	0 (0%)	2 (0.6%)	1
Surgical intervention	0 (0%)	2 (0.6%)	1
Minor	0 (0%)	0 (0%)	1
New conduction disturbances and arrhythmias			
High degree conduction disturbance			
Type 3 paroxysmal AVB	0 (0%)	3 (0.9%)	1
Type 3 permanent AVB	8 (9%)	5 (1.4%)	< 0.01
Other high‐degree conduction disturbance^a^	2 (2.2%)	2 (0.6%)	0.19
New onset AF or flutter	1 (1.1%)	12 (3.5%)	0.48
Permanent pacemaker implantation	22 (25%)	61 (18%)	0.13
AKI	2 (2.2%)	5 (1.4%)	0.59
AKI 3	0 (0%)	1 (0.3%)	
AKI 2	1 (1.1%)	1 (0.3%)	
AKI 1	1 (1.1%)	3 (0.9%)	
Paravalvular regurgitation ≥ 2	9 (10%)	28 (8.1%)	0.54
Moderate and severe PVR	0 (0%)	0 (0%)	1
Mild PVR	9 (10%)	28 (8.1%)	0.54

Abbreviations: AF, atrial fibrillation; AKI, acute kidney injury; AVB, atrioventricular block; PVR, paravalvular regurgitation; TIA, transient ischemic accident.

^a^ includes: 3rd degree AVB (permanent or paroxysmic), syncopal 3rd degree sino‐atrial block, and LBBB/RBBB alternance.

Table A2

**Table A3:** Pre‐index TAVR procedure ECG and new conduction disturbance after TAVR.

	Simplified group (*n* = 89)	Standard group (*n* = 343)	*p*
Pre‐index procedure			
Type 1 AVB	20 (22%)	52 (15%)	0.11
RBBB	14 (16%)	44 (13%)	0.5
LBBB	13 (15%)	40 (12%)	0.46
LAHB	20 (22%)	51 (15%)	0.094
New high‐degree conduction disturbances	19 (21%)	67 (19%)	0.67
Type 3 paroxysmal AVB	2 (2.2%)	17 (4.9%)	0.39
Type 3 permanent AVB	15 (17%)	46 (13%)	0.38
Other high‐degree conduction disturbance^a^	2 (2.2%)	4 (1.2%)	0.61
New LBBB	29 (33%)	95 (27%)	0.33
Permanent pacemaker implantation	22 (25%)	61 (18%)	0.13
Permanent 3rd degree AVB	15 (17%)	46 (13%)	0.38
Other high‐degree conduction disturbance	2 (2.2%)	4 (1.2%)	0.61
New persistent > 48 h LBBB > 150 ms	3 (3.4%)	6 (1.7%)	0.4
Paroxysmal 3rd degree AVB with prior RBBB	2 (2.2%)	1 (1.1%)	0.2
Paroxysmal 3rd degree AVB without prior RBBB	0 (0%)	4 (1.2%)	0.59
Timing of implantation (days)	1 [1; 5]	2 [1; 4]	0.68
Type of valve among patients with new conduction disturbance, *n* (%)	41 (46%)	140 (40%)	0.83
Medtronic	9 (22%)	33 (24%)	
Edwards	32 (78%)	107 (76%)	
Type of valve among patients with new high‐degree conduction disturbance only, *n* (%)	19 (21%)	67 (19%)	0.53
Medtronic	4 (21%)	19 (28%)	
Edwards	15 (79%)	48 (72%)	

*Note:* Data are expressed as a number (%).

Abbreviations: AVB, atrioventricular block; LAHB, left anterior hemiblock; LBBB, left bundle branch block; RBBB, right bundle branch block.

^a^ Includes: syncopal sino‐atrial 3rd degree block (*n* = 2) and LBBB/RBBB alternance (*n* = 4).

Table A3.
